# Leptin Increases: Physiological Roles in the Control of Sympathetic Nerve Activity, Energy Balance, and the Hypothalamic–Pituitary–Thyroid Axis

**DOI:** 10.3390/ijms24032684

**Published:** 2023-01-31

**Authors:** Davide Martelli, Virginia L. Brooks

**Affiliations:** 1 Department of Biomedical and NeuroMotor Sciences, 40126 Bologna, Italy; 2 Department of Chemical Physiology and Biochemistry, Oregon Health & Science University, Portland, OR 97239, USA

**Keywords:** energy expenditure, brown adipose tissue, obesity, diet-induced thermogenesis, sex differences, arcuate nucleus, paraventricular nucleus, obesity-induced inflammation, selective leptin resistance, weight regain

## Abstract

It is well established that decreases in plasma leptin levels, as with fasting, signal starvation and elicit appropriate physiological responses, such as increasing the drive to eat and decreasing energy expenditure. These responses are mediated largely by suppression of the actions of leptin in the hypothalamus, most notably on arcuate nucleus (ArcN) orexigenic neuropeptide Y neurons and anorexic pro-opiomelanocortin neurons. However, the question addressed in this review is whether the effects of increased leptin levels are also significant on the long-term control of energy balance, despite conventional wisdom to the contrary. We focus on leptin’s actions (in both lean and obese individuals) to decrease food intake, increase sympathetic nerve activity, and support the hypothalamic–pituitary–thyroid axis, with particular attention to sex differences. We also elaborate on obesity-induced inflammation and its role in the altered actions of leptin during obesity.

## 1. Introduction

Energy balance is defined as food or energy intake minus energy expenditure, which in turn is determined by internal work (energy expended by normal organ function, which is dependent on ATP formation and ultimately results in heat) plus external work (skeletal muscle activity). If food intake exceeds energy expenditure, because energy cannot be created or destroyed (first law of thermodynamics), the excess energy is stored largely as fat in adipose tissue. However, in young to middle-aged healthy individuals, body weight remains remarkably constant over the long-term (months to years), despite widely varying food intakes and energy expenditures [1,2,3]. Clearly, homeostatic mechanisms must exist to ensure a constant body weight, which, ignoring changes in total body water content, is proportional to fat content. This constancy makes teleological sense from an evolutionary perspective, as a low fat content limits energy reserves during starvation and impedes reproduction, whereas a high fat content impairs predatory survival.

While the mechanisms that underlie the control of food intake and energy expenditure in the short-term (meal-to-meal) are fairly well understood, the mechanisms contributing to the precision of long-term body weight control remain largely unresolved, with one key exception: decreased levels of the hormone leptin, produced in and released from adipose tissue in direct proportion to adipose size, signal starvation to the body, leading appropriately to hunger and reduced energy expenditure [4,5]. Nevertheless, the normal long-term stability of fat mass and body weight suggests that mechanisms must also exist to suppress food intake and increase energy expenditure when the energy balance becomes positive (albeit for relatively short but sustained periods). However, whether increases in leptin levels physiologically signal an adipose surfeit to suppress food intake and increase energy expenditure is often disputed [5]. One argument against such a role is that acutely large, non-physiological doses of exogenous leptin are required [5,6].

The purpose of this review was to consider evidence for and against a role for increases in endogenous leptin in the long-term control of energy balance. Although leptin circulates in plasma, the vast majority of its actions are mediated via binding to receptors in the brain [4]. After central neuronal binding, leptins can influence the function of many organ systems in addition to those involved in the regulation of energy balance, including the hypothalamic–pituitary–thyroid (HPT) axis and the cardiovascular system [4]. The effects of leptin on energy balance as well as on the cardiovascular system are mediated at least in part by changes in the activity of autonomic nervous innervation of organs important in the control of energy balance [4]. Therefore, we particularly focus on its actions to increase sympathetic nerve activity (SNA).

## 2. Dissimilar Impact of Decreases versus Increases in Leptin

The discovery of leptin and the leptin receptor (LepR) pivoted on the generation of two mouse models exhibiting extreme obesity: the *ob/ob* mouse (carrying mutations in the gene responsible for the production of leptin) and the *db/db* mouse (carrying mutations in the gene responsible for the production of LepR). With its discovery, it quickly became clear that decreased leptin levels signal a negative energy balance (predominantly via the JAK-Stat pathway [4]), largely mediated by reduced binding of leptin to its receptors in the hypothalamus [4,5]. Hence, leptin is often called the “starvation hormone.” On the other hand, normal plasma leptin concentrations are near a “threshold” level in most individuals, such that the relationships between leptin and its actions are steep when leptin levels fall, as during fasting, but further elevations in leptin seem to have minimal effects [5,6,7,8]. Explanations for these reduced effects include maximal receptor occupancy at physiological leptin concentrations and, following leptin binding to its receptor, activation of short-loop negative feedback systems via increased levels of the negative cellular regulator, suppressor of cytokine signaling 3 (SOCS3) [9,10]. Nevertheless, it is important to note that most studies documenting the relative resistance to increases in leptin levels utilized large, supraphysiological, and often acute doses of leptin. Do these data, therefore, preclude a role for increases in leptin within the physiological range?

Early research suggests not. Halaas et al. [11] convincingly demonstrated that long-term (2–4 week) subcutaneous leptin infusions in rats, which produced an increase in plasma leptin concentrations by as little as 40%, significantly decreased body weight by both suppressing food intake and increasing energy expenditure. How is this possible if maximal receptor occupancy already exists at normal plasma leptin concentrations? Recent data highlights one explanation. With time (hours to days), leptin induces the expression of its own receptor in two key hypothalamic sites, the arcuate nucleus (ArcN) and the paraventricular nucleus (PVN) [12,13]. Leptin also induces the expression of its receptor in cultured microglia [14]. Thus, sustained increases in leptin could effectively increase its threshold level. While increases in most hormones typically trigger a negative feedback decrease in receptor expression, the ability of leptin to increase the expression of its receptor makes teleological sense in the light of leptin’s role in the long-term control of energy balance. Given, as indicated above, that leptin’s acute effects are limited in part by LepR expression, LepR upregulation due to sustained increments in leptin levels (signaling energy or adipose excess) could unveil its latent anorexic and energy-expending actions.

Recent work suggests that the cellular actions of leptin on brain neurons may also be amplified with time via synergism with other neurotransmitters or neuromodulators. An example from our work: we investigated whether and how leptin in PVN increases SNA and blood pressure, despite a scarcity of LepR. Bilateral PVN injections increased lumbar SNA (LSNA), which innervates hindquarter skeletal muscle, as well as heart rate, blood pressure, and brown adipose tissue (BAT) SNA, but the increases developed slowly, reaching significance only after several minutes [12]. Interestingly, the slowly developing responses were associated with increased LepR expression (see above). These results were consistent with previous studies demonstrating that intracerebroventricular (ICV) leptin administration also slowly increased SNA and blood pressure [15,16] and that prior ICV administration of leptin amplified the hypertension elicited by a second leptin challenge [17]. Further studies revealed that the leptin-induced sympathoexcitatory response depended in part on glutamate, likely via synergism with leptin directly on PVN pre-sympathetic neurons, as previously shown in the hippocampus [18], as well as possibly via the activation of glutamatergic interneurons.

## 3. Physiological Significance of Increases in Leptin

### 3.1. Neurocircuitry by Which Leptin Increases SNA and Energy Expenditure (Figure 1)

Before considering the data for and against a physiological role for increases in leptin in long-term energy balance, it is necessary to outline the brain sites and pathways by which leptin not only inhibits food intake and increases energy expenditure (via both BAT SNA and the HPT axis), but also influences the function of the cardiovascular system.

**Figure 1:**
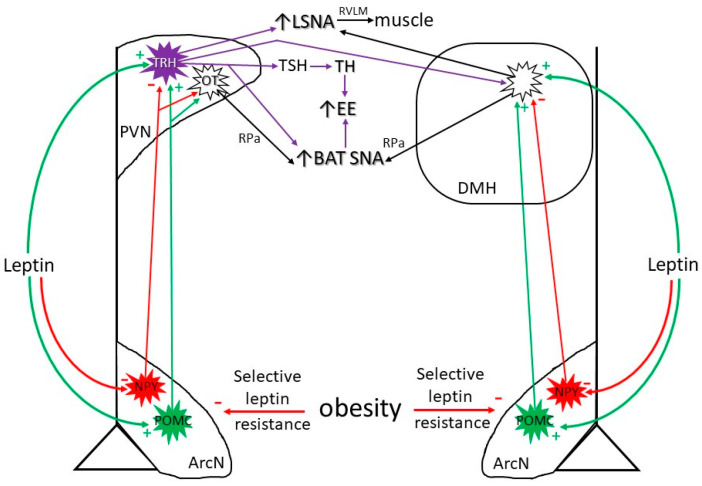
Hypothalamic neurocircuitry by which leptin increases SNA and activates the HPT axis. NPY: Neuropeptide Y; POMC: Pro-opiomelanocortin; ArcN: Arcuate nucleus; PVN: Paraventricular nucleus; TRH: Thyrotropin-releasing hormone; TSH: Thyroid-stimulating hormone; TH: Thyroid hormone; OT: Oxytocin; EE: Energy expenditure; BAT SNA: Brown adipose tissue sympathetic nerve activity; RVLM: Rostral ventrolateral medulla; RPa: Raphe pallidus; DMH: Dorsomedial hypothalamus. Green arrows: stimulatory; red arrows: inhibitory. See text for description and details.

It is well established that the ArcN is a central hub mediating the varied effects of leptin on food intake and energy expenditure. Within the ArcN, key neuronal subtypes include the orexigenic neuropeptide Y/Agouti-related protein (NPY/AgRP) neurons and their counterpart, anorexic pro-opiomelanocortin (POMC) neurons [4,5] (Figure 1). NPY neurons and the downstream neurocircuitry react quickly to stimulate food intake; however, POMC neurons and downstream sites are only slowly engaged, particularly in the context of the control of food intake [5,19]. Since hormones such as leptin excite POMC neurons, this slow responsiveness is again consistent with leptin’s role in counteracting long-term accrual of excess body fat. In turn, ArcN NPY and POMC neurons course to multiple hypothalamic sites, most notably the PVN and DMH [19,20].

Unlike the control of food intake, in which the ArcN is a major site of action, leptin binds to receptors in several hypothalamic sites to increase the activity of sympathetic nerves innervating many organs (kidney, muscle, gut, heart, BAT). Nevertheless, the ArcN again appears to be primary, as ArcN lesions prevented the effect of intravenous (IV) leptin to increase SNA [21] (Figure 1). Further evidence indicates that the PVN is also crucial in the downstream neurocircuitry (Figure 1). More specifically, the sympathoexcitatory response to ICV leptin (which presumably can access many hypothalamic sites [22]) was abolished by nonselective blockade of the PVN [16], also suggesting that the PVN is a site of confluence, regardless of where leptin is binding to initiate increases in SNA. Consistent with this model, ArcN NPY neuronal fibers that project to the PVN tonically inhibit presympathetic neurons reaching the brainstem cardiovascular center, the rostral ventrolateral medulla (RVLM) [23,24], as well as the brainstem center regulating BAT SNA, the raphe pallidus (RPa) [25] (Figure 1). In parallel, ArcN POMC neurons project to and excite presympathetic neurons that increase LSNA (which innervates skeletal muscle) [16,24] and BAT SNA [26] via release of alpha-melanocyte-stimulating hormone (α-MSH) (Figure 1). Importantly, reversal of tonic NPY sympathoinhibition is required before α-MSH can stimulate PVN presympathetic neurons [24].

The PVN contains at least four subsets of pre-sympathetic neurons that project to the brainstem or preganglionic neurons in the spinal cord: those expressing corticotropin releasing hormone (CRH), thyrotropin-releasing hormone (TRH), vasopressin, or oxytocin (OT) [12,27,28]. Increased leptin levels inhibit ArcN NPY neurons and stimulate POMC neurons, thereby increasing LSNA, splanchnic SNA (SSNA), and BAT SNA [12,24,29] via both TRH and OT presympathetic neurons [12,25,30,31] (Figure 1). Leptin’s actions on ArcN NPY and POMC neurons that project to the PVN also inhibit food intake [32]. Finally, considerable information indicates that NPY neurons inhibit and POMC neurons stimulate the release of TRH from PVN neurons that instead progress to the median eminence, where thyroid-stimulating hormone (TRH) is released into the blood to stimulate the production of thyroid hormone (TH) [33,34] (Figure 1). Therefore, leptin can increase energy expenditure by both increasing TH levels as well as BAT SNA.

In addition to the ArcN, leptin is also capable of direct stimulatory actions on PVN TRH neurons, despite a paucity of LepR [12,33,34] (Figure 1). These actions likely rely on LepR receptor induction and synergism with glutamate [12], as described above. Direct leptin stimulation of TRH neurons not only leads to increased production of TH, but it also increases SNA to skeletal muscle (LSNA) and BAT [12], thereby reinforcing the effects of ArcN leptin to increase energy expenditure via these same pathways.

A third hierarchical site of leptin action is the DMH, not only via direct actions [12,35,36,37] but also possibly via indirect actions by suppression of ArcN NPY neurons [20,25] and stimulation of ArcN POMC neurons that project to the DMH [26,38] (Figure 1). As in the PVN, these actions lead to increases in LSNA and BAT SNA.

### 3.2. Diet-Induced Increases in Leptin

Given that leptin can induce the expression of its own receptor, are increments in leptin levels physiologically significant? Leptin-induced increases in LSNA could serve, over a longer time frame than insulin, to stimulate glucose uptake into muscle [39,40], whereas increases in BAT SNA generate heat and increase energy expenditure, both of which are appropriate actions in the face of increased body energy content, such as after eating. Thus, one physiological paradigm that has been extensively studied is diet-induced thermogenesis (DIT). It is important to emphasize, however, that while DIT was first hypothesized to play a role in long-term energy balance, the term DIT has more recently been used to describe the increases in body temperature and energy expenditure that occur as a result of eating a single meal. To distinguish between these two phenomena with widely differing time frames, we refer to the latter as “meal-induced thermogenesis” or MIT.

MIT results from the thermic effects of the digestion and absorption of food, largely of protein and carbohydrates; storage of excess food stuffs increases energy expenditure, but not heat (for reviews, see refs. [41,42,43,44]). In addition, while somewhat controversial due to the varying indirect approaches used to measure BAT activity, considerable evidence suggests that eating leads to BAT activation in both rodents and humans, in part mediated by increased BAT SNA (for reviews, see ref. [41,43,44]). Leptin increases BAT SNA and BAT thermogenesis [45] and leptin levels increase after eating; therefore, does leptin contribute to MIT? In humans, plasma leptin levels rise hours after eating [46,47], emphasizing its role in the long-term control of energy balance, but the rise occurs only after MIT has subsided, thus disputing a role of leptin in MIT. On the other hand, a recent study showed that re-feeding starved (48 h) rats increased body temperature and energy expenditure [48], and in parallel, leptin levels increased from low to normal. Further experiments revealed that the rise in body temperature, which was mediated by BAT activation (prevented by blockade of SNA or removal of BAT) but not an increase in energy expenditure, was dependent on leptin. Nevertheless, in this experimental paradigm, leptin levels increased from below normal back to normal, which again emphasizes the important physiological role of low to normal leptin plasma concentrations (although, notably, increases in leptin to twice normal levels evoked further increases in body temperature in this study). Thus, current evidence supports a major role for leptin in MIT or MIT-induced BAT activation only when its levels are low, as during fasting.

On the other hand, Rothwell and Stock’s groundbreaking work suggested that leptin is critical to the increase in BAT-induced thermogenesis (DIT) that occurs in response to long-term overeating. In their experiments, rats fed a cafeteria diet to induce voluntary hyperphagia exhibited minimal body weight gain because of reduced feeding efficiency due to BAT activation, heat dissipation, and increased energy expenditure [49,50]. Evidence supporting a major role for leptin in increased heat production was that reduced feeding efficiency and DIT were not observed in *ob/ob* mice [51,52]. When humans were overfed for short periods, they also exhibited a compensatory increase in energy expenditure (independent of the increase in body mass) [5,53]. However, it is unknown whether this long-term DIT in overfed humans depends on leptin, although a case has been made for its existence [19,50]. On the other hand, a recent study suggested that another, unidentified, catabolic factor released from adipose tissue inhibited food intake and reduced feeding efficiency in mice overfed for days via gastric infusion [54]. Nevertheless, a role for leptin was not eliminated, since mice in which leptin levels were clamped below normal ate more after release from overfeeding than mice in which leptin levels were allowed to increase with fat mass. Therefore, current evidence implicates at least a partial role for leptin in DIT, but clearly more work is required; in particular, long-term challenge studies in humans need to be conducted.

## 4. Obesity-Induced Increases in Leptin

As indicated above, in the steady-state, energy (food) intake equals energy expended plus storage primarily in adipose tissue. Therefore, storage = food intake − (internal heat + external work). This relationship is the basis of the general consensus that obesity (excess storage) “is the consequence of small, cumulative imbalances between energy intake and expenditure” [5,55]. Nevertheless, some researchers have hypothesized that obesity could instead originate from derangements in food-partitioning or storage mechanisms ([56], see also ref. [5,57]). If so, then in order for obesity to occur, again there must also be derangements in the mechanisms that signal increased adiposity to the brain (such as leptin) and nullification of the subsequent, homeostatic, long-term control of food intake and/or expenditure that would tend to counteract increased adiposity. Therefore, in either case, there must be changes in these homeostatic mechanisms to ultimately allow small persistent increments in adipose to transpire in non-obese individuals. What are these changes? Many explanations have been suggested, including inherited, multi-modal genetic mutations or epigenetically modified genes, as well as environmental factors such as dietary composition, physical activity level, or social and hedonistic influences [5,58]. As an example of the latter, it is well known that many people gain weight during holiday periods [59] and that this excess weight can be sustained [60].

Regardless of how the obese state evolves, it is well established that excess adiposity yields the expected increases in plasma leptin levels. Nevertheless, the elevated leptin levels clearly fail to effectively suppress food intake or increase energy expenditure due to “leptin resistance” [5] (Figure 1). Thus, the excess adiposity comes to be considered normal. In other words, since increased leptin levels normally mitigate against the long-term accrual of body fat, there must be a factor that counteracts this normal response such that there is a shift in the threshold or “setpoint” of body weight maintenance to a higher leptin level. A hotly debated question is: why? One mechanism to explain the reduced effectiveness of elevated plasma leptin levels to counteract increased adiposity is that the transport of leptin from plasma to the brain is attenuated [61,62]. Second, while LepR-expressing hypothalamic neurons critical in the regulation of food intake and energy expenditure retain responsiveness to leptin in obese animals [10,63], responses were reduced with ICV administration of leptin, which bypassed the blood-brain barrier (BBB) transport mechanism [22,61,62]. Thus, leptin still bound and activated its receptor; however, the degree of activation and/or downstream signaling was less than predicted based on leptin levels alone. As a result, ArcN NPY levels were relatively increased and POMC levels were decreased [64]. What then attenuates the responsiveness of hypothalamic LepR to plasma leptin?

### Obesity Suppresses Leptin’s ArcN Anorexic Actions

As described above, one mechanism that normally tends to minimize leptin’s inhibition of food intake and increase in energy expenditure is near maximal LepR occupation, although this limitation can be overcome with time by leptin’s ability to induce the expression of its own receptor. Therefore, obesity could also reduce leptin responsiveness or shift the leptin setpoint by hindering leptin induction of its own receptor. In support of this view, while obesity has been shown to increase LepR expression in the ArcN [10,13], the administration of leptin to further increase plasma levels in obese mice failed to induce additional LepR expression, unlike in lean mice [13]. Thus, leptin-induced increased LepR expression appears to be restrained during obesity, thereby contributing to a reduction in leptin’s actions.

With obesity, elevated levels may also suppress the ability of leptin to inhibit food intake via induction of cellular signaling negative feedback mechanisms, as described above, such as SOC3 and the tyrosine phosphatase, PTP1B, both of which are increased, specifically in the ArcN [10,65]. A recent study proposed that leptin’s ability to mute its own actions via this feedback is a major contributor to leptin resetting [10,65] and that small decreases in leptin levels could actually unmask its anorexic actions. However, if so, why is weight loss in obese individuals, which is accompanied by a decrease in plasma leptin levels, so difficult to maintain (i.e., the fall in leptin levels causes hunger and reduced energy expenditure rather than reduced hunger; see below)?

In addition to limits in hypothalamic LepR expression and induction of cellular negative feedback signaling pathways, significant evidence indicates that obesity also induces hypothalamic inflammation, microglia activation, and cytokine production [64,66], which correlates with and contributes to ArcN leptin resistance [5]. The inflammation associated with obesity is a classic example of a low-grade systemic response affecting the whole body, brain included [67]. This low-grade, sustained over time, inflammatory response has also been termed metabolic inflammation or meta-inflammation [68]. We will now describe immune derangements, beginning first with peripheral inflammation and then the occurrence and mechanisms of central inflammation.

## 5. Obesity: A State of Inflammation

### 5.1. Obesity-Induced Systemic Inflammation

Adipose tissue is directly involved in immune function. Adipocytes and macrophages have many characteristics in common; indeed, pre-adipocytes can differentiate into macrophages [69]. White adipose tissue (WAT) has the ability to release more than 600 different bioactive molecules, including cytokines and chemokines, which are collectively known as adipokines [70,71,72] (Table 1).

The concept that obesity is associated with a low-grade inflammatory state stems from demonstrations that circulating levels of interleukin-6 (IL-6) and C-reactive protein (CRP), both pro-inflammatory markers, are elevated in obese animals and humans [105], while they decrease back to normal levels after weight loss [84]. In addition, obesity can be associated with an increase in the number of circulating neutrophils and other white blood cells (WBCs) but a specific decrease in cytotoxic T cells and NK cells [106,107]. The inflammation can be widespread: Fischer and colleagues showed that a high fat diet (HFD) for 20 weeks induced increases in body weight, glucose intolerance, and inflammation in the liver, perigonadal fat, and subcutaneous fat in mice. Most of these parameters returned to normal after 7 weeks of a low fat diet (LFD); however, inflammation persisted in the liver and perigonadal fat, which becomes critical in the context of sustained weight loss (see below) [108].

The low-grade systemic inflammatory status associated with obesity likely evolves from several sources [71]. As previously reviewed in detail [55,109], the number of adipocytes is set or locked in parallel with a low level of constant turnover in humans after the age of about 20. As a result, as lipid storage needs and adiposity increase, the excess lipid requires an enlargement of resident adipocytes. In turn, adipose cell growth necessitates a remodeling of the extracellular matrix (ECM) to support the larger fat cells, ultimately leading to fibrosis [109]. In addition, the increased adipocyte size causes hypoxia, oxidative stress [110], and the active secretion of cytokines. These adverse changes trigger infiltration of leukocytes into the adipose tissue [70,109]. The adaptive immune remodeling of WAT, in part induced via the recruitment of WBC by the chemokine, CxCl12 [111], parallels the increased expression of pro-inflammatory markers in adipose tissue [112]. Hypertrophic adipocytes can also eventually burst, thereby worsening the local inflammatory response [70]. WAT inflammation can therefore trigger a vicious cycle through which other organs become inflamed and the inflammation emanating from local sites yields increased systemic inflammation. Obesity can also induce an increase in gut permeability, which if exaggerated, might lead to endotoxemia [i.e., increased levels of lipopolysaccharide (LPS)] [113,114,115]. Finally, obesity is associated with an increase in SNA to the muscle and kidney (see below) [116,117]. Sympathetic nerves are a double-edge sword when we consider their role in the control of immune function: they promote anti-inflammatory effects during the course of a systemic infection [118,119], but they also have the ability to mediate pro-inflammatory effects when chronically overactivated [120,121], as is the case in obese people. Sympathetic nerve activation in WAT is also associated with enhanced non-esterified fatty acids and cytokine loads in the adipose microenvironment, including that of pancreatic fat, which likely impair beta-cell function and contribute to insulin resistance [122].

### 5.2. Obesity-Induced Neuroinflammation

Obesity triggers inflammatory responses in several areas of the CNS, including the hypothalamus, mediated by microglia and astroglia activation and proliferation. Importantly, hypothalamic inflammation first identified in preclinical studies has also been detected in obese humans [123,124]. Multiple mechanisms are likely involved. The first description of obesity-induced hypothalamic inflammation came from the work of De Souza and colleagues [125]. These authors showed that a high fat diet (HFD) for 4 months produced high levels of IL-1β, TNF, and IL-6 in the hypothalamus of rats, mediated through the JNK and NF-kB pathways. This was later confirmed in obese mice subjected to a much more restricted period of HFD (24–72 h) [123]. In the short term, saturated fatty acids (SFAs), especially long chain SFAs, bond to TLR-4 and triggered the classic pro-inflammatory physiological response [126].

The hypothalamic inflammatory response, therefore, can precede systemic inflammation and the establishment of obesity, suggesting a possible causal role for neuroinflammation in initiating a pathophysiological response that leads to obesity. Several studies support this scenario: (1) Specific and selective deletion of IKKB, a key protein involved in the intracellular inflammatory cascade response, in hypothalamic astrocytes reduced the susceptibility of mice to diet-induced obesity [127]. (2) In rats fed a HFD, ICV administration of IL-4, a key regulator in humoral and adaptive immunity, further increased HFD-mediated hypothalamic inflammation and caused even more excessive weight gain. If the rats were treated with an IKKB–NF-kB blocker, body weight and fat mass decreased [128]. (3) The inhibition of microglial expansion in the ArcN limited food intake with subsequent decreases in body weight and fat content in mice [129]. (4) One to three days of HFD increased the expression of the chemokine, CX3Cl1, in the hypothalamus of mice that were obesity-prone but not in those that were obesity-resistant. Inhibition of CX3Cl1 reduced the glucose intolerance and fat content of obesity-prone mice without affecting their body weight [130]. (5) Five days of HFD induced an overexpression of CXCl12 and its receptors, CXCR4 and CXCR7, both in the PVN and lateral hypothalamus (LH). Hypothalamic administration of CXCl12 in normal diet (ND)-fed animals recapitulated the effects of HFD consumption; it: (i) reduced novelty-induced locomotor activity, (ii) increased encephalin gene expression in the PVN, and (iii) induced an overconsumption of HFD rather than ND if the rats had a choice. Collectively these responses contributed to weight gain [131].

With chronic obesity, a second contributor to hypothalamic inflammation may be peripheral inflammation. Several adipokines, such as leptin, as well as cytokines, can cross the BBB to activate microglia [71,132]. Other potential, but to our knowledge currently unproven, scenarios include: (1) HFD rapidly alters the gut microbiome and increases gut permeability, leading to leakage of bacterial LPS into the blood [114,115] and activation of brain microglia [133,134]. Microglia activation may be direct, as LPS or its components has been shown to bind to brain endothelial cells and tanocytes and enter the brain, possibly via a lipoprotein transport mechanism [135]. Interestingly, LPS can also activate iNOS, which has been shown to trigger the transformation of ArcN perivascular macrophages into microglia-like immune cells, which move into the brain parenchyma [136]. Alternatively, SFA or gut bacteria (or their byproduct, LPS) may activate vagal afferents [137,138], which can increase hypothalamic inflammation [139]. Intriguingly, LPS, like obesity, has been shown to cause leptin resistance via induction of the negative cellular regulator, PTP-1B [140]. (2) Systemic inflammation can lead to disruption of the BBB, promoting leukocyte extravasation and increasing diffusion of solutes across the BBB, including the entry of pathogens and toxins in the brain parenchyma. This has the ability to trigger a vicious cycle responsible for inducing further inflammation [141].

### 5.3. Potential Actions of Leptin to Facilitate Inflammation with Obesity

The most direct evidence for a role of leptin in establishing the low-grade systemic inflammatory response associated with obesity is that WAT inflammation was strongly reduced when LepR were knocked out in mouse leukocytes [76]. Leptin also directly stimulates T cells, macrophages, and neutrophils [73] and is essential for normal T-cell proliferation; its deficiency caused thymus atrophy and severe immune dysfunction [73,74]. In addition to systemic proinflammatory actions, leptin may also enable central inflammation. For example, by binding to brain endothelial receptors, leptin promoted neutrophil migration to and into the brain following LPS administration [132,142]. Indeed, obesity, via increased leptin levels, exaggerated the neuroinflammatory response to LPS administration (for review, see ref. [71]). However, to our knowledge, a specific role for increased leptin levels in facilitating or mediating brain inflammation during obesity has not been established.

## 6. Obesity Induces Selective Leptin Resistance

The information summarized above suggests that, in obese individuals, elevated leptin levels fail to adequately suppress food intake or increase energy expenditure (i.e., leptin resistance) due to hypothalamic inflammation, maximal LepR occupancy, and decreased LepR responsiveness secondary to cellular negative feedback inhibition. However, much of the extensive research on this topic has been conducted without specific attention to potential sex differences. It is well-established that plasma leptin levels are higher in females than in males, either lean or obese. The pioneering studies of Clegg and colleagues [143] also documented that females are more responsive to the anorexic actions of leptin than males due to the sensitizing effects of estrogen. Nevertheless, the fact that women, like men, become obese despite elevated leptin levels suggests that resistance to the anorexic actions of leptin do develop, although preclinical studies suggest that a longer time on a HFD is required [144,145]. Clearly, more work is needed.

While, especially in males, the anorexic responses to leptin dissipate with obesity, leptin’s ability to influence other systems remains intact or is even enhanced, giving rise to so called “selective leptin resistance.” This selectivity is apparent on at least two levels: (1) within the ArcN, obesity suppresses the effect of leptin to inhibit food intake but not its control of other modalities; and (2) leptin’s ability to suppress food intake and trigger appropriate signaling mechanisms that underlie the regulation of many modalities are muted in the ArcN but not in other leptin-receptive hypothalamic sites. As with the anorexic actions of leptin, sex differences have also been observed. We will address two of leptin’s effects that elude the development of resistance: leptin-induced activation of cardiovascularly relevant SNA and the HPT axis, including what is currently known about sex differences.

### 6.1. Selective Leptin Resistance in the ArcN: Preserved Leptin-Induced Increases in SNA (in Obese Males)

The concept of selective leptin resistance was first identified in the context of leptin-induced activation of the sympathetic nervous system and its contribution to obesity-induced hypertension. As nicely summarized in their review [146], Mark and colleagues were the first to show that leptin’s actions to suppress food intake and increase energy expenditure were suppressed in genetic mouse models of obesity and diet-induced obesity, but its ability to increase renal SNA (RSNA) was preserved or enhanced. Head and colleagues similarly reported that in HF diet-induced obese compared to lean rabbits, ICV administration of leptin induced larger increases in RSNA but smaller increases in c-fos in many hypothalamic regions, including the ArcN and PVN [22], again highlighting the selective preservation of leptin’s sympathoexcitatory mechanisms despite more global cellular resistance.

As recently reviewed [24,147], several lines of evidence indicate that elevated leptin levels in obese males increase SNA and can, if it occurs, contribute to hypertension: (1) increases in MSNA correlate with leptin and adiposity levels in humans [24,148,149,150,151]; (2) nonspecific blockade of the ArcN or PVN decreased SNA and blood pressure in obese rodents [152,153]; and (3) selective blockade of LepR, including specifically in the ArcN, decreased SNA and blood pressure in obese animals [21,154,155]. Recent work has investigated whether changes in the function of ArcN NPY or POMC neurons in males with obesity underlie sensitization to the sympathoexcitatory effects of leptin and/or its metabolic partner, insulin. Briefly, the data suggest (see ref. [24] for a review) that tonic inhibition of PVN presympathetic neurons by NPY is absent in obese males, allowing sympathetic stimulation by leptin and insulin to progress unimpeded [156]. In addition, it appears that simultaneously enhanced signaling mechanisms in ArcN POMC neurons mediate the amplified sympathoexcitation to leptin and insulin [153].

**Figure 2:**
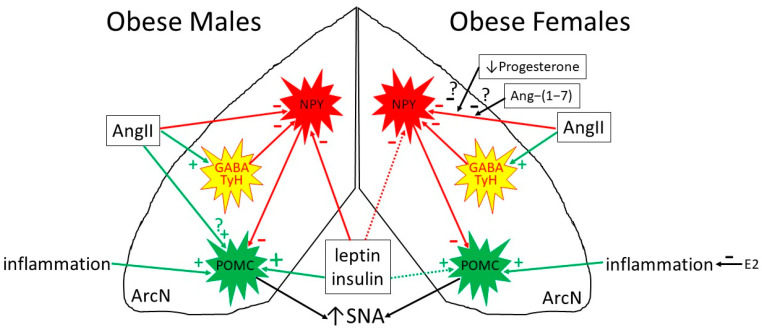
Hypothesized mechanisms underlying sex differences in the actions of elevated leptin and insulin levels to increase SNA during obesity. NPY: Neuropeptide Y; POMC: Pro-opiomelanocortin; ArcN: Arcuate nucleus; AngII: Angiotensin II; Ang-(1–7): Angiotensin 1–7; TyH: Tyrosine hydroxylase (marker of dopaminergic neurons); GABA: Gamma-aminobutyric acid; E2: Estrogen; SNA: Sympathetic nerve activity. Green arrows: stimulatory; red arrows: inhibitory. Dotted arrows indicate that NPY and POMC presympathetic neurons are resistant to the actions of leptin or insulin in obese females, whereas these actions are enhanced in obese males. See text and legend to Table 2 for details. Adapted in part from [24].

One mechanism that has been hypothesized to mediate the select preservation of leptin-induced actions within the ArcN is that different leptin-induced signaling pathways are engaged in resistant versus sensitive neurons [146,157]. Another hypothesized mechanism involves the actions of the renin-angiotensin system (RAS) in the brain, since the sympathoexcitatory and hypertensive actions of leptin require functional AngII type 1 receptors (AT1aR), whereas the anorexic effects apparently do not [24,146] (Figure 2). Similar to leptin and insulin, AngII in the ArcN increased SNA and blood pressure [158,159] in male rats through binding to AT1aR; the downstream pathway depended upon inhibition of NPY and stimulation of POMC neurons that project to the PVN [159]. A detailed neuroanatomical assessment of the location of AT1aR in the ArcN of male rats [159,160] revealed that (1) approximately 10% of AT1aR were expressed on NPY neurons; (2) AT1aR were rarely found on POMC neurons (4%); (3) instead, AT1aR were expressed largely in neurons that co-expressed both tyrosine hydroxylase (TyH; dopaminergic) and VGat2 (GABAergic) (see also ref. [161]); (4) LepR positive neurons rarely co-expressed AT1aR. The distribution of AT1aR in the mouse ArcN differed in that AT1aR were more commonly found in NPY- and LepR-containing neurons, although the paucity of AT1aR in POMC neurons was similar (2%) [162]. Based on information in rats, it was hypothesized that ArcN AngII increases SNA through either direct or indirect (via stimulation of TH/GABAergic interneurons) inhibition of sympathoinhibitory ArcN-to-PVN NPY neurons. This NPY disinhibition allows other stimulatory factors to activate sympathoexcitatory POMC neurons, such as AngII, leptin, insulin, or even inflammation. Obesity activates the RAS both systemically and centrally [163,164,165]. Therefore, one hypothesis to explain the select sensitization of ArcN presympathetic neurons to leptin in obese males is that this very small cohort of sensitized neurons are those that co-express AT1aR with POMC and/or LepR.

The situation in females is quite different. While increases in MSNA correlate with adiposity in men, a similar correlation is generally not observed in women (for reviews, see ref. [24,149,165]). At least two factors may be involved: (1) In lean males, leptin increases the activity of several sympathetic nerves; yet, in lean females, leptin’s stimulation of some sympathetic nerves can vary with the reproductive cycle. More specifically, similar to leptin’s anorexic actions in females [143], leptin-induced increases in LSNA and RSNA require proestrus (heightened) levels of estrogen, whereas increases in SSNA and HR do not vary during the estrus cycle and are similar between males and females [15]. Therefore, the dependence of leptin on elevated estrogen levels to increase lumbar (preclinical studies) or muscle (humans) SNA may lead to cycle-mediated variability that disrupts any correlation. (2) Strikingly, unlike in obese males in which the sympathoexcitatory responses to insulin or leptin can be enhanced (see above), insulin [156] and leptin (Shi and Brooks, unpublished data) fail to increase SNA in obese females; in other words, obese females come to exhibit leptin and insulin resistance rather than sensitization to its sympathoexcitatory actions. Investigations of the mechanism revealed that tonic PVN NPY sympathoinhibition is not reduced in obese females as it is in obese males, but it is unchanged [156]. More importantly, the tonic NPY sympathoinhibition was not inhibitable (by insulin), thereby imposing an irreversible blockade of sympathoexcitatory inputs.

In obese females, several possible (but unproven) mechanisms may resist the sympathoexcitory effects of leptin or insulin [24,163,166] (Figure 2): (1) POMC neurons were never found to co-express AT1aR, preventing this direct route of activation; (2) AT1aR expression is induced by progesterone, as during estrus or pregnancy (when ArcN AT1aR levels can become very high), but obesity tends to decrease progesterone levels; (3) obesity in females is not associated with central inflammation; and (4) obesity in females activates the anti-hypertensive arm of the RAS, Ang-(1-7), which could counteract AT1aR-mediated sympathoexcitation and hypertension, specifically in the ArcN.

### 6.2. Preserved or Enhanced Leptin Responsiveness in the PVN and DMH: Leptin Support of the HPT Axis and of BAT SNA with Obesity

In lean animals and humans, fasting suppresses the HPT axis and energy expenditure via a concurrent decrease in plasma leptin levels through reversal of leptin’s tonic inhibition of ArcN NPY neurons and excitation of ArcN POMC neurons that project to PVN TRH neurons [33,34,167]. The direct effect of leptin on PVN TRH neurons is not involved, which is not surprising, given the marked paucity of LepR in the PVN compared to that in the ArcN. As described above, obesity renders ArcN NPY and POMC neurons resistant to leptin, including those that influence PVN TRH neurons. However, obese humans and diet-induced obese rats continue to demonstrate normal or even elevated TRH levels and activity of the HPT axis in both males and females [168,169,170,171,172]. At least in males, this enhanced HPT axis activity is instead mediated by the direct actions of leptin to stimulate PVN TRH neurons [168], perhaps through an upregulation of PVN LepR, although this has not been directly tested. With obesity, the direct action of leptin on PVN TRH presympathetic neurons that project to the RVLM may also contribute to hypertension, since knockdown of hypothalamic preproTRH normalized blood pressure in hypertensive diet-induced obese rats [173].

Finally, evidence supporting sustained activation of DMH leptin-receptive neurons was demonstrated in obese mice. More specifically, leptin activated BAT in DIO and *ob/ob* mice in the face of muted anorexic actions. Blockade of LepR in the DMH prevented the activation of BAT by leptin, implicating a role for this hypothalamic site in the sustained thermogenic effects of leptin during obesity [35].

A summary of the sites and mechanisms of select leptin resistance in males versus females is provided in Table 2.

**Table 2:** Selective Leptin Resistance: Summary of Sex differences.

	Males		Females	
	Lean	Obese	Lean	Obese
Plasma leptin levels	_	↑	↑	↑↑
Food intake with increased leptin	↓	_	↓↓ (dependent on estrogen)	_
LSNA with increased leptin	↑	↑↑	↑ (dependent on estrogen)	0
SSNA with increased leptin	↑	?	↑	?
Tonic PVN NPY sympathoinhibition	_	↓(0)	_	_
PVN POMC (α-MSH) sympathoexcitation	_	↑	_	↓
HPT axis	_	_	_	_
HPT axis: support mediated by ArcN leptin	_	↓	_	↓ (?)
HPT axis: support mediated by PVN leptin	0	↑	0 (?)	↑ (?)

**Table 2.** While the increased leptin levels that occur with obesity fail to suppress food intake in both sexes, its actions to increase SNA are increased in males (which can cause hypertension) but nullified in females (obese reproductively active females do not exhibit increased SNA). These sex differences appear to pivot on differential changes in the ArcN. More specifically, obese males exhibit sustained suppression of tonically inhibitory ArcN NPY neurons and activation of POMC neurons that project to the PVN. In addition, the data indirectly suggest that increased drive of PVN presympathetic TRH neurons due to increased expression of PVN LepR and leptin binding may also contribute to hypertension development. In contrast, obesity leads to leptin resistance in ArcN NPY and POMC presympathetic neurons in females. In both sexes, the activity of the HPT axis is maintained or increased. In males, there is evidence that despite the development of resistance to leptin in ArcN NPY and POMC neurons that control the HPT axis, TRH/TSH or thyroid hormone levels are normal or elevated due to increased stimulation of PVN TRH neurons by endogenous leptin, possibly in part because of elevated LepR expression. The symbol “_” denotes the response in lean males to which all other responses are compared. ↑: increased; ↑↑: increased more compared to other ↑ in rows; ↓: decreased; ↓↓: decreased more compared to other ↓ in row; ?: unknown or unclear.

### 6.3. The Role of Leptin in Weight Regain

Multiple studies, including those based on the show “The Biggest Loser,” have clearly documented that the increased body weight of obese individuals is homeostatically defended such that it is nearly impossible to maintain weight loss [5,8,109,174]. While several factors fuel this recidivism [8,109], here we focus on the contribution of sustained leptin resetting. More specifically, elevated leptin levels fall with weight loss, which act inappropriately as a starvation signal to increase hunger and decrease energy expenditure in both men and women [8,174,175,176] due to activation of NPY neurons and inhibition of POMC neuron, in the ArcN (i.e., unremitting leptin resetting) [5,109]. Nevertheless, the activity of systems exhibiting sustained leptin sensitivity with obesity, such as the HPT axis and SNA to the vascular beds of cardiovascularly relevant organs, decreases with weight loss, leading to resolution of hypertension [177,178] and a decrease in TH levels. Thus, the decrease in energy expenditure (relative to body weight) is mediated in part by suppressed activity of BAT as well as of the HPT axis [174,179,180]. Evidence that irreversible leptin resetting and decreasing leptin and TH levels are a major contributor to the inability of formerly obese humans to maintain weight loss is that leptin and/or thyroxine administration increased the indices of SNA and also supported weight loss [181,182,183]. This sustained leptin resetting seems to dispute the hypothesized action of decreases in endogenous leptin (such as with weight loss) to enhance LepR responsiveness via decreases in negative signaling regulators or a role for maximal LepR occupation as major contributors to the leptin resetting with obesity. Therefore, the question then becomes: which mechanisms underlie sustained leptin resetting of the control of food intake in formerly obese individuals? One such mechanism pivots on the failure of obesogenic adipose inflammation to dissipate with a reduction in body weight and adipose tissue.

As described above, adipocytes enlarge with obesity, thereby promoting inflammation. However, as fat cells shrink due to a negative energy balance and weight loss in obese individuals, mechanical stress in the ECM ensues along with maintained fibrosis and accompanying sustained or even augmented adipose tissue inflammation [108,109,184,185,186,187], despite the resolution of systemic and muscle inflammation and improved glucose handling. Importantly, the sustained adipose inflammation is associated with sustained or increased hypothalamic inflammation, as deduced from the expression levels of cytokines and other inflammatory mediators in hypothalamic tissue blocks [188,189,190], as well as immunohistochemical assessment of iba-1, a microglial marker, in the ArcN [64,191].

Additional indirect evidence that unrelenting hypothalamic inflammation mediates sustained leptin resetting during obesity reversal is that the two treatments that most successfully support long-term weight loss and reverse leptin resetting also reverse hypothalamic inflammation: Roux-en-Y gastric bypass (RYGB) [192] and liraglutide [a glucagon-like-peptide 1 (GLP-1) agonist] treatment [64]. Unlike calorie restriction, which causes weight loss in association with increased ArcN orexigenic NPY/AgRP expression and decreased anorexigenic POMC expression, RYGB decreases body weight without changes in these neuromodulators [190,193,194,195]. In parallel, RYGB decreases hypothalamic indices of inflammation [190,192,196] and increases leptin sensitivity [192,194]. However, while RYGB can decrease adipose inflammation, this has not been a consistent finding [197,198,199]; therefore, are there other mechanisms that might explain how RYGB reverses hypothalamic inflammation? Current hypotheses stem from changes in gut function [195]. First, modification of the intestinal microbiome may reduce systemic metabolic endotoxicity, which is known to impact the hypothalamus in such varied conditions as cancer-induced cachexia and autonomic responses to bacterial toxemia [192]. Second, levels of GLP-1, a peptide released from the small intestine, are significantly increased after RYGB [195] and exert anti-inflammatory effects [200]. As mentioned above, liraglutade is a GLP-1 agonist currently being tested as an obesity reversal treatment [201,202]. Liraglutide not only inhibits or reverses ArcN microglial activation in obese subjects losing weight [64,191], but it also appears to suppress inhibitory PTP1B signaling, thereby enhancing leptin sensitivity [191,203] in part by direct actions on POMC and NPY neurons [204]. Collectively, these actions reverse leptin resetting and support sustained weight loss. Thus, sustained hypothalamic inflammation with weight loss secondary to calorie restriction correlates with sustained leptin resetting, but further work is required to mechanistically establish this link.

## 7. Summary and Future Research Directions

It is well-established that decreased plasma leptin levels, as with fasting, signal starvation and elicit appropriate physiological responses, such as increasing the drive to eat (and the intake of food, if available) and decreasing energy expenditure. These responses are mediated by suppression of the actions of leptin in the hypothalamus, most notably on ArcN orexigenic NPY neurons and anorexic POMC neurons. However, the main question addressed in this review is whether the effects of increased endogenous (or exogenous) leptin levels are also physiologically significant on the long-term control of energy balance and the sympathetic nervous system, despite conventional wisdom to the contrary. To summarize, we suggest the following:

While eating increases leptin levels, the rise is delayed relative to MIT; therefore, it appears that leptin is not a major player in meal-evoked thermogenesis, except in fasting individuals. On the other hand, DIT occurs over a much longer time frame and helps to limit weight gain despite chronic overeating. Current evidence suggests that increases in leptin levels contribute, but also that other unidentified hormonal mechanisms are involved.

Obesity also increases plasma leptin levels. While failing to decrease food intake due to ArcN leptin resistance or resetting (secondary to hypothalamic inflammation, maximal LepR binding, and negative feedback mechanisms), increased leptin levels exert other significant effects. In both sexes, elevated leptin levels support the HPT axis, likely via increased actions in the PVN, possibly due to the induction of LepR expression in TRH neurons. Particularly in obese males, increased leptin levels increase SNA to the muscle and kidneys, thereby contributing to hypertension development. In sharp contrast, obese females exhibit leptin (and insulin) resistance and are less likely to develop hypertension secondary to sympathoexcitation. Obesity engenders a low-grade inflammatory state, which may also depend, in part, on increased leptin levels. Lastly, a major early but disappointing finding was that exogenous leptin administration failed to reverse obesity. Nevertheless, preliminary investigations suggest that leptin administration to individuals undergoing weight loss, when leptin levels fall due to sustained hypothalamic inflammation and leptin resetting, may help sustain weight loss. Agents that reverse hypothalamic inflammation, such as GLP-1, may also be beneficial.

Nevertheless, much remains to be discovered. Here, we suggest a few questions that, when answered, could yield high impact. (1) Do increased leptin levels contribute to obesity- or DIT-induced increases in energy expenditure (the HPT axis or BAT SNA) or hypertension via induction of its receptor in PVN or ArcN (if so, which cell type)? Does leptin induce its receptor in other brain areas? Leptin is a cytokine: what are the roles of LepR in microglia or astroglia to aid and abet obesity-induced hypothalamic inflammation? (2) What are the contributions of LPS, vagal afferent stimulation, and leptin in obesity-induced neuroinflammation? (3) Does select reversal of hypothalamic inflammation after weight loss reduce or eliminate weight regain? (4) What other catabolic hormones contribute to homeostatic BW regulation with sustained increases in food intake and/or decreases in energy expenditure? (5) How is adipose or systemic inflammation transmitted to the CNS? (6) Does anti-immune therapy with or without leptin treatment [55,205] or treatment with a GLP-1 agonist [201,202] help sustain weight loss? Minocycline has been successfully used to reverse or prevent hypothalamic microglial activation in rodents fed a high fat diet [206,207]; does this or a similar agent help sustain weight loss? (7) Finally, studies focusing on sex differences and their mechanisms are lacking. In this context, studies of the brain RAS and its role in energy balance, obesity, or obesity-induced increases in SNA and hypertension development are clearly needed.

## Figures and Tables

**Table 1:** Obesity-induced cytokine responses.

Cytokine	Description	Changes with Obesity
Leptin	Pro-inflammatory adipokine released by fat cells. Stimulates T cells, macrophages, and neutrophils to release pro-inflammatory cytokines [73]. Leptin is essential for normal T-cell proliferation and its deficiency causes thymus atrophy and severe immune dysfunction [73,74]. Surprisingly, leptin improves survival during sepsis [75].	Levels directly related to fat stores; increase with obesity. WAT inflammation was strongly reduced when LepR was knocked out in leukocytes in DIO mice [76].
Adiponectin	An anti-inflammatory adipokine that modulates a number of metabolic processes [77].	Adiponectin circulating levels are usually inversely proportional to the level of visceral adiposity; therefore, obese individuals have very low levels of adiponectin [78]. The adiponectin–leptin ratio is often considered a functional marker of inflammation associated with obesity [77].
Resistin	Also known as adipose tissue-specific secretory factor, it plays a role in the pathogenesis of atherosclerosis by enhancing the synthesis of hepatic LDL [79].	Elevated with non-morbid obesity. Acts locally on leukocytes, located in the WAT, to induce the release of pro-inflammatory cytokines [80].
Tumor necrosis factor α (TNF)	TNF is a necessary and sufficient mediator of inflammation, acutely released by macrophages, T cells, and natural killer cells during infection [81]. TNF is also released by adipocytes [82].	Its plasma levels are generally high in obese individuals, especially in those presenting visceral obesity rather than subcutaneous obesity [83]. However, it is not clear whether its levels decrease after weight loss [84].
IL-6	A proinflammatory cytokine produced by immune, endothelial, and muscle cells as well as adipocytes [70]. Surprisingly, an anti-IL-6 antibody therapy, used for the treatment of rheumatoid arthritis, causes weight gain [85]	Plasma levels are correlated with BMI and especially with adipose tissue mass [86]. Surgery- induced weight loss is associated with a significant decrease in IL-6 levels [87].
Monocyte chemoattractant protein-1 (MCP-1)	Key chemokine that regulates migration and infiltration of monocytes and macrophages [88].	Circulating MCP-1 levels do not differ between lean and obese individuals [89]; however, levels of this chemokine increase selectively in the WAT of obese adults [90]. MCP-1 levels in plasma significantly decrease after Roux-en-Y Gastric Bypass (RYGB) surgery or a low calorie diet [70,91].
Interleukin 8 (IL-8)	Pro-inflammatory and chemoattractant cytokine [92].	Systemic levels are closely correlated with BMI, waist circumference, and other obesity-related parameters [93]. Weight loss is not always associated with a decrease in IL-8 plasma levels; in fact, low calorie diet-induced weight loss is associated with an increase in IL-8 levels [94], whereas weight loss induced by RYGB produces a decrease in IL-8 levels [95].
Interleukin 10 (IL-10)	Anti-inflammatory cytokine released by M2 macrophages, Th2 T cells, neutrophils, and adipocytes [96].	Systemic IL-10 levels are generally inversely correlated with BMI and body fat percentage [70,97]. Nevertheless, plasma IL-10 levels have been reported to be elevated in obese women; however, obese women with lower IL-10 levels were more prone to develop metabolic syndrome [98].
C-reactive protein (CRP)	Released by hepatocytes in response to trauma, infection, or injury [99].	CRP levels are significantly higher in obese individuals than in lean subjects [86] and decrease with diet or weight loss induced by surgical intervention [100,101], making it a good marker of meta-inflammation.
Transforming growth factor β (TGF-β)	Cytokine released by all leukocytes [102].	Regardless of the location of fat mass, obesity is associated with enhanced levels of TGF-β [103], while weight loss decreases TGF- β circulating levels [104].

## Data Availability

Not applicable.
